# 1156. Real-World Effectiveness of Recombinant Zoster Vaccine in Chinese Adults Aged ≥50 Years in the US

**DOI:** 10.1093/ofid/ofad500.996

**Published:** 2023-11-27

**Authors:** Ana Florea, Lina S Sy, Lei Qian, Bradley Ackerson, Yi Luo, Jun Wu, Yanjun Cheng, Jennifer H Ku, Leticia I Vega Daily, Harpreet Takhar, Jeannie Song, Elizabeth Chmielewski-Yee, O M areen Spence, Harry Seifert, Driss Oraichi, Hung Fu Tseng

**Affiliations:** Kaiser Permanente Southern California, Pasadena, CA; Kaiser Permanente Southern California, Pasadena, CA; Kaiser Permanente Southern California, Pasadena, CA; Kaiser Permanente Southern California, Pasadena, CA; Kaiser Permanente Southern California, Pasadena, CA; Kaiser Permanente Southern California, Pasadena, CA; Kaiser Permanente South California, San Gabriel, California; Kaiser Permanente Southern California, Pasadena, CA; Kaiser Permanente, Northridge, California; Kaiser Permanente - Southern Calfornia Research And Evaluation - EPI, Pasadena, California; Kaiser Permanente Southern California, Pasadena, CA; GSK, Rockville, Maryland; GSK, Rockville, Maryland; GSK, Rockville, Maryland; GSK, Rockville, Maryland; Kaiser Permanente Southern California, Pasadena, CA

## Abstract

**Background:**

Following the May 2019 approval of recombinant zoster vaccine (RZV) in China, there was a need for real-world information on the vaccine effectiveness (VE) of RZV in a Chinese population. We conducted a retrospective cohort study at Kaiser Permanente Southern California (KPSC) to estimate the VE of two doses of RZV in preventing herpes zoster (HZ) and chart-confirmed post-herpetic neuralgia (PHN) in Chinese adults.

**Methods:**

The study cohort consisted of Chinese KPSC members aged ≥50 years who received two doses of RZV 4 weeks to ≤6 months apart; second doses were accrued from 6/1/2018 to 12/31/2020. Chinese individuals were identified based on self-reported ethnicity or self-reported spoken/written language (Chinese, Mandarin, Cantonese, Taishanese, Taiwanese, Hmong, Shanghainese). RZV unvaccinated Chinese individuals were matched 4:1 to their vaccinated counterparts by age and sex, with follow-up through June 2022. The index date for vaccinated individuals was the date of the second dose; unvaccinated individuals had the same index date as their vaccinated match. Cox proportional hazards models were used to estimate adjusted hazard ratios (aHRs) with 95% confidence intervals (CIs) comparing outcomes (HZ and PHN) in the vaccinated and unvaccinated groups. Adjusted VE (%) was calculated as (1−aHR) × 100.

**Results:**

3978 RZV vaccinated Chinese individuals were matched to 15,912 RZV unvaccinated Chinese individuals. We identified 15 HZ cases in the vaccinated group and 344 in the unvaccinated group, with incidence rates per 1000 person-years (95% CI) of 1.5 (0.9–2.5) and 10.9 (9.8–12.1), respectively. There were 0 PHN cases in the vaccinated group and 19 in the unvaccinated group. The models were adjusted for heart disease, immunocompromised status, number of outpatient/virtual visits, history of zoster vaccine live vaccination, and length of continuous membership. Adjusted VE (95% CI) was 87.6% (78.9–92.7) against HZ; VE against PHN could not be estimated due to a lack of PHN cases. A sensitivity analysis excluding 30 days after index date from follow-up yielded similar results.

**Conclusion:**

Among Chinese adults aged ≥50 years, two doses of RZV provided substantial protection against HZ and PHN, supporting the real-world VE in this population.

**Disclosures:**

**Ana Florea, PhD MPH**, Gilead: Grant/Research Support|GlaxoSmithKline: Grant/Research Support|Moderna: Grant/Research Support|Pfizer: Grant/Research Support **Lina S. Sy, MPH**, Dynavax: Grant/Research Support|GlaxoSmithKline: Grant/Research Support|Moderna: Grant/Research Support **Lei Qian, PhD**, Dynavax: Grant/Research Support|GlaxoSmithKline: Grant/Research Support|Moderna: Grant/Research Support **Bradley Ackerson, MD**, Dynavax: Grant/Research Support|Genentech: Grant/Research Support|GlaxoSmithKline: Grant/Research Support|Moderna: Grant/Research Support|Pfizer: Grant/Research Support **Yi Luo, PhD**, GlaxoSmithKline: Grant/Research Support|Moderna: Grant/Research Support|Pfizer: Grant/Research Support **Jennifer H. Ku, PhD MPH**, GlaxoSmithKline: Grant/Research Support|Moderna: Grant/Research Support **Leticia I. Vega Daily, MSW**, GlaxoSmithKline: Grant/Research Support **Jeannie Song, MPH**, GlaxoSmithKline: Grant/Research Support|Moderna: Grant/Research Support|Pfizer: Grant/Research Support **Elizabeth Chmielewski-Yee, PhD, MPH**, GSK: Employee|GSK: Stocks/Bonds **OMareen Spence, PhD**, GSK: Employee|GSK: Stocks/Bonds **Harry Seifert, MD. MSCE, FISPE**, GSK: Employee|GSK: Stocks/Bonds **Driss Oraichi, PhD**, GSK: Employee|GSK: Stocks/Bonds **Hung Fu Tseng, PhD MPH**, GSK: Grant/Research Support|Moderna: Grant/Research Support

